# Resistance to Amino Acid Biosynthesis Inhibiting-Herbicides in *Amaranthus palmeri* Populations from Aragon (Spain)

**DOI:** 10.3390/plants14101505

**Published:** 2025-05-17

**Authors:** Eneko Trebol-Aizpurua, Mikel V. Eceiza, Clara Jimenez-Martinez, Ana I. Marí, Mercedes Royuela, Ana Zabalza, Miriam Gil-Monreal

**Affiliations:** 1 Institute for Multidisciplinary Research in Applied Biology (IMAB), Universidad Pública de Navarra (UPNA), Campus de Arrosadia, 31006 Pamplona, Spain; trebol.132420@e.unavarra.es (E.T.-A.);; 2 Department of Forestry and Agricultural Science and Engineering, University of Lleida and AGROTECNIO Center, Av. Rovira Roure 191, 25198 Lleida, Spain; 3 Department of Plant Protection, Integrated Pest Management Group, Centro de Investigación y Tecnología Agroalimentaria de Aragón (CITA), 50059 Zaragoza, Spain

**Keywords:** target-site, nicosulfuron, imazamox, glyphosate, *ALS* mutation, *EPSPS* gene amplification

## Abstract

*Amaranthus palmeri* is a highly problematic agricultural weed due to its rapid growth, high seed production, and strong tendency to develop herbicide resistance. In Spain, the initial colonization of *A. palmeri* began in 2007, when populations were detected at various locations in the province of Lleida (Catalonia). Since then, new infestations have been reported in other regions of the country, primarily infesting maize fields. Although resistance to glyphosate or to acetolactate synthase (ALS) inhibitors has been documented in several populations from Catalonia and Extremadura, little is known about the resistance profile of populations from Aragon. The main objective of this study was to characterize the putative resistance of five populations from Aragon to 5-enolpyruvylshikimate-3-phosphate synthase (EPSPS) inhibitors (glyphosate) and ALS inhibitors (nicosulfuron and imazamox). Sensitivity to both mechanisms of action was measured by root growth in vertical plates and shikimate accumulation for glyphosate. Target-site resistance was evaluated by analyzing *EPSPS* and *ALS* gene copy numbers and *ALS* gene mutations. The populations showed high variability, with no multiple resistance detected. The Bujaraloz population showed moderate resistance to glyphosate due to *EPSPS* gene amplification. In three populations, mutations in the *ALS* gene conferring resistance were detected. The Trp574Leu mutation was detected in approximately half of the individuals from the Albelda, Tamarite de Litera, and Caspe populations. In the latter, the Pro197Thr mutation was also present. This study reveals significant genetic variability within each population and provides evidence for the spread of herbicide resistance across different regions of Spain.

## 1. Introduction

Palmer amaranth (*Amaranthus palmeri* S. Watson) is a dicotyledonous summer annual weed that is considered to be one of the most troublesome weeds of warm-season crops, such as soybean (*Glycine max* (L.) Merr.), maize (*Zea mays* L.), cotton (*Gossypium hirsutum* L.), and sorghum (*Sorghum bicolor* (L.) Moench) [1,2,3,4]. Several biological characteristics make *A. palmeri* extremely competitive, including its C4 photosynthetic pathway, rapid growth rate, high fecundity, extended emergence period, and high stress tolerance [5,6]. As a dioecious obligate outcrossing species, *A. palmeri* has high genetic variability that has contributed to the evolution of its resistance to multiple herbicides [5]. Currently, *A. palmeri* has been reported to possess resistance to herbicides with nine different mechanisms of action, including acetolactate synthase (ALS) inhibitors and 5-enolpyruvylshikimate-3-phosphate synthase (EPSPS) inhibitors [7].

Herbicides are a dominant tool for weed control in conventional cropping systems, even though non-chemical methods are also an indispensable component of Integrated Weed Management (IWM) strategies. Glyphosate is the most widely used herbicide globally, and although it was developed as a broad-spectrum herbicide, it has been used as a selective herbicide since the introduction of glyphosate-resistant crops in the mid-1990s [8]. Glyphosate is the only herbicide that inhibits EPSPS, a key enzyme in the aromatic amino acid biosynthetic pathway [9].

ALS inhibitors were introduced in the early 1980s and are among the most popular post-emergence herbicides. These herbicides are important due to their high crop selectivity, low application rates, and relatively low environmental impact [10]. They act by blocking the action of the first enzyme in the biosynthesis of branched-chain amino acids [11]. ALS inhibitors are a large herbicide group with 59 active ingredients divided into six chemical families, with sulfonylureas and imidazolinones being the most important.

The over-usage of herbicides contributes to the development of herbicide-resistant weed populations. EPSPS and ALS inhibitors are among the herbicide classes with the most resistant weed populations detected [7]. In recent years, glyphosate resistance has increased rapidly, reaching a total of 60 species worldwide [7]. Meanwhile, currently, 176 weed species have at least one population that is resistant to ALS inhibitors, making them the herbicide group with the greatest number of resistance cases [7]. The most aggressive species, such as *A. palmeri*, have hundreds of resistant populations reported around the world. Herbicide resistance can be conferred by two main types: target site (TSR), related to the target enzyme itself, and non-target site (NTSR). In *A. palmeri*, TSR and NTSR mechanisms have been described for both EPSPS and ALS inhibitors.

In the case of glyphosate, the most frequent TSR mechanism is *EPSPS* gene amplification [12,13,14,15], resulting in an exceptionally high accumulation of the EPSPS protein [16]. Point mutations in the *EPSPS* gene that alter the structure of the target enzyme have also been found in some *A. palmeri* populations [17]. Regarding NTSR mechanisms, reduced absorption and translocation have been documented in some *A. palmeri* biotypes [17,18,19].

For ALS inhibitors, the most common TSR mechanism in *A. palmeri* involves point mutations in the *ALS* gene, which prevents herbicides from binding to the target while preserving the enzyme’s functionality [20,21,22,23]. Meanwhile, detoxification via cytochrome P450 monooxygenases (P450s) is the main NTSR mechanism for ALS inhibitors identified in *A. palmeri* [21].

Although *A. palmeri* is native to northern Mexico and southern California [24], it has expanded well beyond its original native range, with populations now present in North and South America, Europe, Asia, and the wider Mediterranean region, and with many of the detected populations showing resistance to herbicides [22,25,26,27,28]. A recent study on several populations from Turkey reported resistance to glyphosate, likely due to *EPSPS* copy number variations, as well as resistance to ALS inhibitors through point mutations [29]. In Israel, mild glyphosate resistance was observed in *A. palmeri* populations collected from agricultural fields, while resistance to ALS inhibitors was confirmed in four populations, most likely associated with the Trp574Leu mutation [30].

In Spain, the initial stages of colonization of *A. palmeri* occurred in 2007, when populations of *A. palmeri* were detected at two locations in the province of Lleida (Catalonia) along the margins of a maize field and roadsides [31]. Subsequent reports of *A. palmeri* infestations have been confirmed in other regions of the country, including additional areas in Catalonia, as well as Aragon and Extremadura. The presence of several *A. palmeri* biotypes resistant to herbicides have already been confirmed in Spain. The first cases of *A. palmeri* resistance to ALS-inhibiting herbicides in Spain were documented in 2020, with resistance linked to multiple amino acid substitutions in the ALS enzyme at positions Pro197, Trp574, and Ser653 [32]. Another study on ALS-inhibiting herbicides in *A. palmeri* populations revealed that resistance is linked to three distinct mutant alleles in the *ALS* gene (Pro197Thr, Asp376Glu, and Trp574Leu) [33]. Additionally, a recently discovered *A. palmeri* population is likely to be resistant to glyphosate by *EPSPS* gene amplification [34].

The aim of the present work was to characterize the TSR mechanisms to glyphosate and ALS-inhibiting herbicides in five *A. palmeri* populations from roadsides and crop fields in Aragon (Spain), where herbicide control failures have been reported. In this region, glyphosate is extensively used in pre-planting desiccation applications in maize, the main crop affected by *A. palmeri*, particularly under no-tillage systems, where glyphosate is the primary tool for weed control before sowing. Glyphosate is also the main herbicide used for roadside vegetation management, typically alternating with mowing, with little use of alternative herbicides. Post-emergence weed control in maize fields relies heavily on the ALS-inhibitor nicosulfuron, which not only targets monocotyledonous weeds but also affects some *Amaranthus* species. Seeds were collected from plants that had survived repeated glyphosate applications on roadsides and from individuals persisting in crop fields exposed to multiple years of herbicide treatments, typically involving four to five active ingredients, with nicosulfuron being the most frequently used. The populations were analyzed to determine their resistance profiles and to identify the underlying TSR mechanisms, providing essential information for the development of effective *A. palmeri* management strategies.

## 2. Results and Discussion

The resistance pattern and potential TSR mechanisms of the five *A. palmeri* populations were assessed by first evaluating their response to glyphosate, followed by testing their possible resistance to two ALS inhibitors: nicosulfuron and imazamox.

### 2.1. Resistance to Glyphosate

#### 2.1.1. Glyphosate Dose–Response Bioassays in Vertical Plates

**Figure 1:**
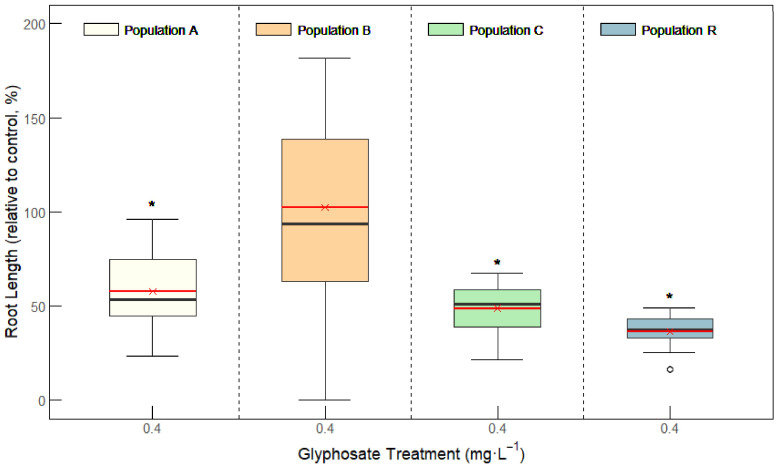
Root length in populations A, B, C, and R of *Amaranthus palmeri* from Aragon. Box plots show 1st, 2nd, and 3rd quartiles; the minimum and maximum; and the mean (red line) (n = 15–20). Root length (Y axis) is expressed as percentage of non-treated samples of each population. For each population, significant differences between treated roots (0.4 mg L^−1^ of glyphosate) and non-treated are highlighted with * (Student’s *t* test; *p* value < 0.05).

Among the four populations evaluated, only population B showed no sensitivity to glyphosate, with its root length reaching 98% of the control (Figure 1). In contrast, root growth in populations A, C, and R was inhibited in length by 43%, 52%, and 66%, respectively, compared to their untreated controls (Figure 1). These results indicate that populations A, C, and R were sensitive to glyphosate.

#### 2.1.2. Hydroponic Tanks and Shikimate Content

**Figure 2:**
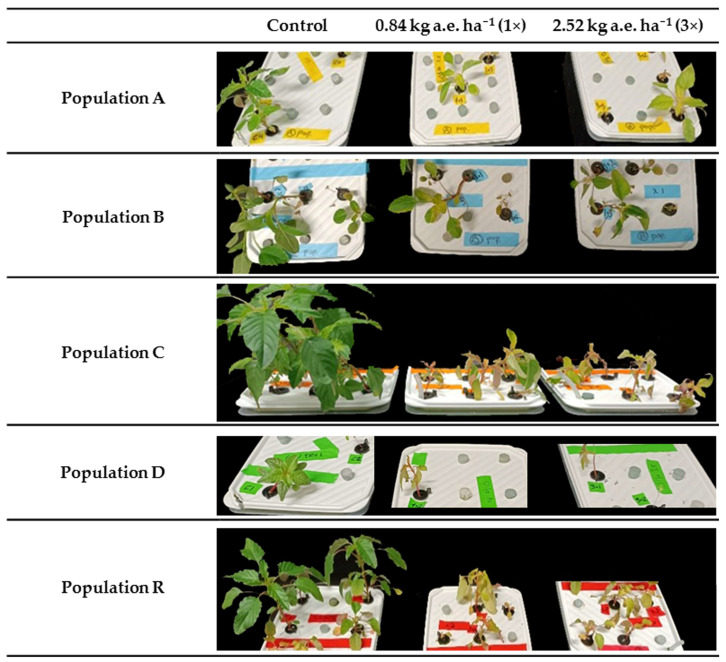
Visual appearance of the five *Amaranthus palmeri* populations (A, B, C, D, and R) 5 days after the treatment: non-treated (control) and glyphosate treatment at the recommended field rate (1×; 0.84 kg a.e. ha⁻^1^) and at three times the recommended field rate (3×; 2.52 kg a.e. ha⁻^1^). For each treatment, 12 plants were used.

Untreated plants of different populations grown in hydroponic tanks showed a high variability in size and vigour. In the case of population D, the germination rate was very low (around 30%), even after the application of gibberellic acid. Five days after glyphosate treatment, treated plants from populations A, C, D, and R showed dose-dependent symptoms, like chlorosis and growth arrest, consistent with previous reports for sensitive populations [16,23]. In contrast, treated plants from population B showed only mild chlorosis and maintained apical growth (Figure 2). All treated plants (12 plants per treatment in each population) from populations A, C, D, and R died within approximately seven to ten days after treatment. However, around 60% and 50% of the 1× and 3× field rate-treated plants, respectively, from population B remained alive after 19 days, although they were visibly affected (Figure S1).

**Figure 3:**
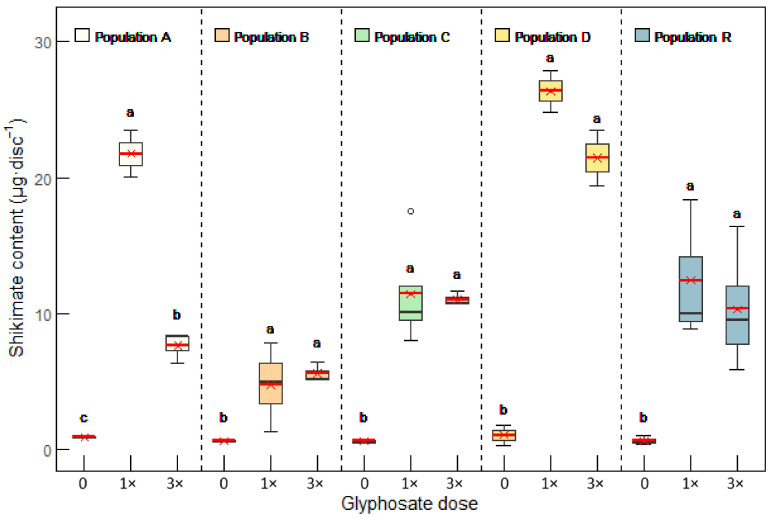
Shikimate content in leaves of populations A, B, C, D, and R. Plants were untreated (control, 0) or sampled 5 days after treatment with glyphosate, applied at 1× (0.84 kg a.e. ha^−1^) and 3× (2.52 kg a.e. ha^−1^) the field rate. Box plots show 1st, 2nd, and 3rd quartiles; the minimum and maximum; and the mean (red line) (n =4). In each population, different letters indicate significant differences between treatments (ANOVA; HSD Tukey; *p* value < 0.05).

Shikimate accumulation was used as a marker of glyphosate sensitivity, as it reflects EPSPS inhibition and allows for discrimination between resistant and sensitive plants [12,16,23,35] (Figure 3). The shikimate content was very low in the untreated plants of all populations, with average values ranging from 0.6 to 1 μg disc^−1^. Following glyphosate treatment, shikimate levels increased in populations A, C, D, and R, reaching values between 7.6 and 26.4 μg disc^−1^. In population B, the shikimate content also increased but to a lesser extent, with treated plants accumulating up to 5.6 μg of shikimate disc^−1^.

In all populations, the 1× field rate of glyphosate was sufficient to induce shikimate accumulation levels comparable to those observed at the 3× field rate. Surprisingly, in population A, shikimate levels at the 3× dose were lower than those at the 1× dose, likely because the plants were already severely affected. The linear correlation between the glyphosate dose and shikimate content reaches the maximum, and at very high, near lethal doses, the shikimate content can even decrease.

#### 2.1.3. EPSPS Gene Copy Number

**Figure 4:**
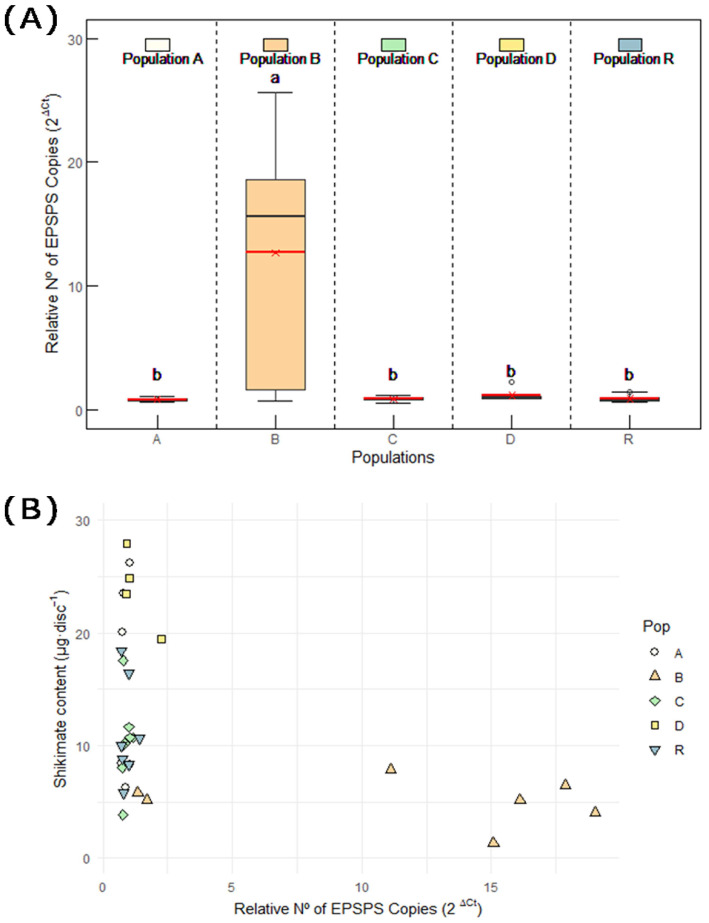
(**A**) *Amaranthus palmeri* genomic copy number of *5-enolpyruvylshikimate-3-phosphate synthase* (*EPSPS*) relative to *carbamoylphosphate synthetase* (*CPS*), expressed as 2^ΔCt^ in the populations of Aragon: A, B, C, D, and R. Box plots show 1st, 2nd, and 3rd quartiles; the minimum and maximum; and the mean (red line) (n = 6–12). Different letters indicate significant differences between treatments (ANOVA; HSD Tukey; *p* value < 0.05). (**B**) Correlation between genomic copy number of *EPSPS* and shikimate accumulation in treated individuals of all populations (Pop). Shikimate accumulation was measured after five days of treatment with 1× (0.84 kg a.e. ha^−1^) or 3× (2.52 kg a.e. ha^−1^) glyphosate field rates.

The genomes of the plants of populations A, C, D, and R exhibited an *EPSPS* mean copy number of 0.9–1.1, while the plants in population B showed gene amplification (Figure 4A). The relative *EPSPS* copy number for population B plants was very variable, with a mean of 12.7 ± 2.7 (Figure 4A). A recent study indicated that it is very likely that the *EPSPS* gene copy number variation is the main mechanism of resistance to glyphosate of a Spanish *A. palmeri* population from Catalonia [34], and our study confirms this TSR mechanism in one population from Aragon. Nevertheless, although *EPSPS* point mutations are not the main TSR mechanism for glyphosate in *A. palmeri*, their coexistence, as well as the involvement or NTSR mechanisms, cannot be ruled out.

The relationship between the shikimate content and the number of *EPSPS* gene copies in the populations was plotted (Figure 4B). No linear correlation was observed between the specific gene copy number and shikimate content. Notably, plants without gene amplification showed a wide range of shikimate values, while individuals from population B (which presented variable gene amplification) clustered at similar low shikimate contents. The lack of a linear correlation between the specific gene copy number and shikimate content might be explained by the inherent variability or might suggest the existence of NTSR mechanisms that would influence shikimate accumulation [36]. Nevertheless, these results support the functional role of *EPSPS* amplification in resistance, since the glyphosate-treated plants of population B accumulated almost no shikimate, consistent with their elevated *EPSPS* copy number, whereas susceptible populations (A, C, D, and R), which lacked gene amplification, showed high shikimate levels.

Gene copy amplification in *A. palmeri* can be up to 160 times higher in resistant populations, while resistant populations of *Bassia scoparia* and *Amaranthus tuberculatus* have fewer duplicated *EPSPS* copies, ranging from four to ten times [15,37]. Nevertheless, despite having fewer duplicated *EPSPS* copies, *A. tuberculatus* populations exhibit resistance levels similar to those of *A. palmeri* populations [38]. The gene amplification level detected in population B was lower than usually detected in *A. palmeri*. Concomitantly, the resistance level was not so high, and treated plants were affected by the herbicide, although they did not die.

The presence of *A. palmeri* in Spain is believed to be the result of contaminated seed or grain imports for feed production [31,32,33,34]. Given the short time between the first detection of *A. palmeri* in Spain and the initial reports of herbicide resistance, it is likely that resistant populations were already resistant upon introduction, rather than evolving locally. Genetic studies by Manicardi and colleagues support this hypothesis, showing that some *A. palmeri* populations invading Spain were resistant prior to their arrival [33,34]. However, the low level of resistance due to *EPSPS* gene amplification observed in population B from Aragon may be attributed to the relatively short (but strong) history of glyphosate selection pressure. Taking this into account, along with the variability in plant responses observed in the hydroponic tank experiment, the results may suggest that resistance is currently evolving in this population.

### 2.2. Resistance to ALS Inhibitors

#### 2.2.1. Dose–Response Bioassays in Vertical Plates

**Figure 5:**
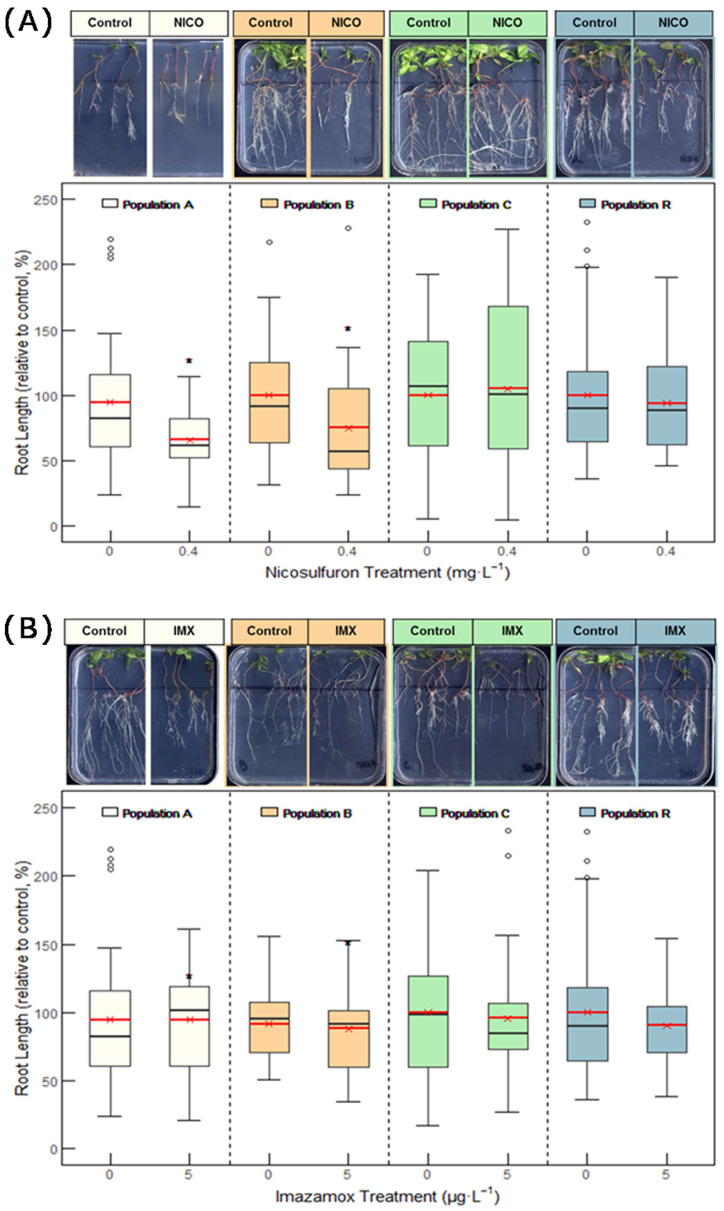
Root length in populations A, B, C, and R of *Amaranthus palmeri* from Aragon, treated with nicosulfuron (**A**) or imazamox (**B**). Box plots show 1st, 2nd, and 3rd quartiles; the minimum and maximum; and the mean (red line) (n = 15–20). Root length (Y axis) is expressed as percentage of non-treated samples of each population. For each population, significant differences between treated and non-treated samples are highlighted with * (Student’s *t* test; *p* value < 0.05). Representative response of each population is shown (top of the figure).

There was considerable variability in the root length among the plants of each population. After nicosulfuron treatment, populations A and B showed growth inhibition (Figure 5A), with root lengths reduced by 34% and 25%, respectively, compared to the untreated controls. In contrast, visual inspection indicated that populations B and R were the most affected by herbicides, with symptoms such as reduced shoot development (Figure 5A). Due to the low seed germination, it was not possible to assess the effect of herbicides on population D in vertical plates. However, hydroponic growth revealed that population D showed mild resistance to sulfonylureas (Figure S2). Imazamox treatment did not result in root growth inhibition in any population, likely due to the high variability in root size and growth rate (Figure 5B).

Resistance to ALS-inhibitor herbicides was evaluated in all populations by the presence of TSR mechanisms: *ALS* gene amplification and *ALS* gene mutations.

#### 2.2.2. ALS Gene Copy Number

**Figure 6:**
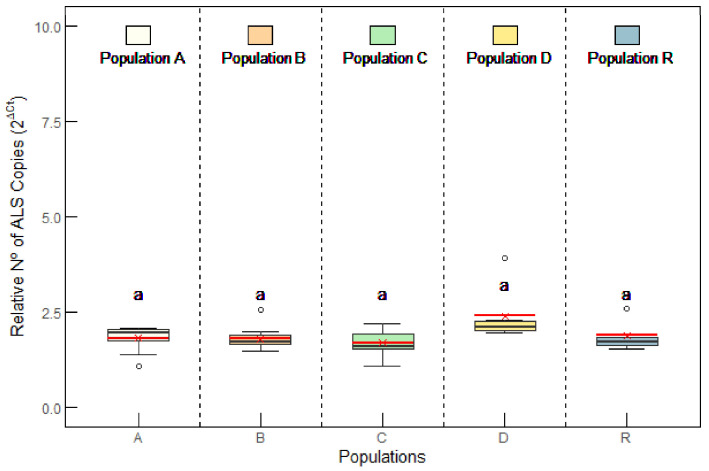
*Amaranthus palmeri* genomic copy number of *acetolactate synthase* (*ALS*) relative to *carbamoylphosphate synthetase* (*CPS*) in populations of Aragon: A, B, C, D, and R. Box plots show 1st, 2nd, and 3rd quartiles; the minimum and maximum; and the mean (red line) (n = 6–10). No significant differences in *ALS* gene copy numbers were detected among populations (ANOVA; *p* value < 0.05).

*ALS* gene amplification, as a mechanism of resistance to ALS inhibitors, is rare in plant species. However, selection pressure with ALS inhibitors may isolate genotypes with increased *ALS* gene copies. This was not the case for the evaluated populations from Aragon, as all populations had similar *ALS* gene copy numbers (about 1.50–2 relative copies; Figure 6), indicating no evidence of gene amplification, as has been reported before for *A. palmeri* [23,39]. Other studies have shown a slight increase in the *ALS* gene copy number in resistant populations of *A. palmeri* and *Alopecurus aequalis* [39,40]. Nevertheless, their main mechanism of resistance was attributed to *ALS* mutations, as has been detected in most populations resistant to ALS inhibitors [41,42].

#### 2.2.3. ALS Mutations

Two populations, B and R, did not show any mutation, while populations A, C, and D showed substitutions at codons associated with resistance to ALS-inhibiting herbicides. In populations A and D, a single amino acid substitution at position 574 (Tryptophan to Leucine) was detected in 50% and 66% of the analyzed plants, respectively (Table 1; Table S1). This substitution was first reported in *A. palmeri* in 2018 [43], and in Spain in 2020 [32], and it is known to confer broad resistance to imidazolinones and sulfonylureas [22,44,45].

In population C, two different substitutions were found. Two-thirds of the plants had the Trp574Leu substitution, as was also the case for Pro197Thr replacement, both found in homozygous and heterozygous forms (Table 1; Table S1). In half of the cases, the Pro197Thr and Trp574Leu mutations were detected in different individuals, while in the other half, both mutations were found in the same plant (Table S1).

There are a total of 11 different amino acid substitutions at position Pro197 that are known to confer resistance to most sulfonylurea herbicides in weeds [7]. In *A. palmeri*, only four substitutions have been reported to date, Pro197Ser, Pro197Ala, Pro197Thr, and Pro197Asp [46], with the first two being the most frequently observed. Although the Pro197Thr substitution identified in this study is common in other species, it has rarely been reported in *A. palmeri*. Interestingly, this amino acid change has also been detected in other *A. palmeri* populations from Spain [33]. It would be interesting to conduct genetic analyses to determine whether these geographically distinct populations carrying the same rare substitution share a common origin. This mutation has been associated with resistance to sulfonylureas but not to imidazolinones [33].

Populations A, D, and C showed target-site mutations at Trp574, and population C also showed mutation at Pro197. The presence of mutations in population C aligns with the observed resistance in the vertical plate assay with nicosulfuron. Similarly, population D exhibited a moderate level of resistance to sulfonylureas (Figure S2), which is consistent with the *ALS* sequencing results. In contrast, root growth in population A was inhibited in the presence of nicosulfuron in the vertical plate assay (Figure 5A), which may be explained by the high proportion of plants lacking TSR mutations in this population.

Populations B and R did not exhibit any mutations in the *ALS* gene, which, in the case of population B, is consistent with the sensitivity observed in the vertical plate assay. In contrast, population R showed neither known mutations in the *ALS* gene, nor an increase in the *ALS* genomic copy number, suggesting the involvement of other resistance mechanisms, such as mutations in undocumented positions or NTSR mechanisms. Further tests should be conducted in order to verify this hypothesis.

The results of the discriminatory test with imazamox do not align with the genetic profile, as only the plants carrying the Trp574 mutation are expected to be resistant to this herbicide. The lack of inhibition across all populations suggests that the imazamox concentration used may have been too low to reliably differentiate resistant and susceptible individuals. Given the variability in root growth among the individuals of the different populations, a higher dose or a full dose–response experiment may be needed to better assess resistance levels. Alternatively, the lack of effects of imazamox may indicate the presence of other resistance mechanisms in these populations, including NTSR or mutations at different positions not analyzed in this study.

A clear correlation between the growth response and genotype was observed in all populations, except for population B, as has been discussed, in the nicosulfuron assay. The amino acid changes found at positions Pro197 and Trp574 indicate that half or more of the tested plants of three populations (A, C, and D) in Aragon were resistant to ALS inhibitors. A similar percentage (35–55%) of amino acid substitutions was previously reported in plants analyzed from northeastern Spain [32]. Changes in positions Pro197 and Trp574 are expected to persist in these populations, since mutations at these points do not represent a major fitness cost [42].

## 3. Materials and Methods

### 3.1. Plant Material

Seeds of *Amaranthus palmeri* were originally collected in the provinces of Huesca and Zaragoza, from the region of Aragon, located in northeastern Spain. Several populations of *A. palmeri* have been identified in Spain in recent years, raising concerns over their potential impact on local cropping systems. These populations were collected from diverse agroecological zones, including agricultural fields and roadsides (Table 2), and from plants that survived repeated glyphosate or nicosulfuron applications, respectively.

Seeds were collected at the time of optimum maturity by cutting the inflorescences and allowing them to dry at room temperature. Once the plant material was dried, it was sieved to obtain the seeds. The sample selected for the experiments contained only shiny, black-colored seeds of hard consistency, eliminating broken, non-uniform, and hollow seeds. The samples were kept in a freezer at −4 °C until their use.

### 3.2. Nicosulfuron and Imazamox Dose–Response Bioassay with Vertical Plates

To monitor herbicide effects, *A. palmeri* seedlings of populations A, B, C, and R were grown on vertical agar plates, and root growth was measured, as has been conducted for *A. palmeri* with other herbicides, such as metolachlor [47,48], and in other species with glyphosate [49]. Seeds were surface-sterilized prior to germination [50] and placed on agar plates containing different treatments. The medium was prepared as previously described [49], with some modifications. The solution consisted of a half-strength Murashige and Skoog (MS) Basal Salt Mixture (Sigma-Aldrich, Saint Louis, MO, USA) and 0.9% (*w*/*v*) agar, adjusted to a pH of 5.8–6. Sterile square vertical dishes (120 mm per side; Simport, Bernard-Pilon Beloeil, Canada) were used. Between eight and ten seeds were placed on each treatment plate (three plates per treatment) at a distance of 3.5 cm from the edge of the plate. Plates were sealed with hypoallergenic microporous paper tape and placed vertically in a growth chamber for 48 h for a 16 h/8 h day/night cycle at 30 °C/18 °C. After germination, the plates were transferred to a phytotron under controlled conditions (day/night cycle of 18 h/6 h; light intensity, 500 μmol s^−1^ m^−2^ PAR; temperature, 24 °C/18 °C; relative humidity, 60/70%).

Preliminary dose–response experiments were conducted using a reference-sensitive population to determine appropriate rates of glyphosate and nicosulfuron that reliably discriminate between sensitive and resistant individuals (Figure S3). The imazamox dose used in this study was selected based on previous works [49]. Fortin Green^®^ (glyphosate isopropilamine salt, 36%; Key, Tárrega, Spain) was applied at 0.4 mg a.i. L^−1^. As ALS inhibitors, sulfonylurea nicosulfuron and imidazolinone imazamox were applied. Talisman^®^ (nicosulfuron, 4% *w*/*v*; Key, Tárrega, Spain) was applied at 0.4 mg a.i L^−1^. Pulsar 40^®^ (imazamox, 4% *w*/*v*; BASF, Barcelona, Spain) was applied at 0.005 mg a.i. L^−1^.

After 10–15 days (depending on the growth rate of each population), when most of the untreated roots had reached the end of the agar plates, the plates were scanned. Root length was measured using ImageJ version 1.53. Root growth is expressed as a percentage relative to the untreated (control) plants within each population. Analyses were performed using at least 15–20 biological replicates from different individual plants. The experiment using vertical plates was performed twice.

### 3.3. Plant Growth in Hydroponic Tanks and Glyphosate Application

Plants were cultivated and grown hydroponically [16]. Seeds were incubated in the dark for 48 h at 4 °C and then maintained for a 16 h/8 h day/night cycle at 30 °C/18 °C until germination. After germination, seedlings were transferred to aerated hydroponic tanks in a phytotron (day/night cycle of 16 h/8 h; light intensity, 500 μmol s^−1^ m^−2^ PAR; temperature, 24 °C/18 °C; relative humidity, 60/70%). For population D, seeds were sprayed with gibberellins (0.25 g L^−1^) prior to their transfer to the phytotron to enhance germination. As in previous studies, all plants were treated with glyphosate at the field rate (1×) and three times the field rate (3×) [51]. Glyphosate is recommended at 0.84 kg a.e. ha^−1^ [52], and the commercial formulation Fortin Green^®^ (glyphosate isopropilamine salt, 36%; Key, Tárrega, CAT, Spain) was used. Herbicide application was carried out using an aerograph (Junior Start model, Definik, Sagola, Spain) connected to a compressor (Werter One, Beverrato). Plants were treated at the growth stage BBCH14 [53]. Each hydroponic tank was divided into two halves, with each considered an experimental plot to which a treatment was applied. Each half contained four plants, and three replicates were performed per treatment.

Five days after herbicide application, photographs of the plants were taken to assess the phenotypic effects of the treatments, and four individual plants were sampled per treatment in each population. For shikimate determination, three leaf disks (4 mm in diameter) were excised from the youngest leaf of both treated and non-treated individuals and stored at −80 °C until use. The remaining leaves from the same individual plants were collected together in the same vial and immediately frozen in liquid nitrogen. Samples were ground to a fine powder under liquid N_2_ using a Retsch mixer mill (MM200, Retsch, Haan, Germany) and stored at −80 °C until they were used for genomic DNA extraction.

### 3.4. Shikimate Content Determination

Shikimate was extracted as previously described, and its content was quantified spectrophotometrically [16].

### 3.5. Genomic DNA Extraction

Genomic DNA (gDNA) was extracted from approximately 0.1 g of previously ground *A. palmeri* leaf tissue, as previously described [16]. The DNA concentration was quantified using a Synergy HT microplate reader (Biotek Instruments, Vermont, USA). The DNA quality was assessed by 1% agarose gel electrophoresis. The xtracted gDNA was used for the quantification of genomic *EPSPS* and *ALS* copy numbers and for *ALS* gene sequencing.

### 3.6. EPSPS and ALS Gene Copy Number

To evaluate the resistance mechanism, the genomic copy number of *EPSPS* and *ALS*, relative to the endogenous single-copy control gene that encodes the large subunit of carbamoylphosphate synthetase (*CPS*) (EC 6.3.5.5), were measured by quantitative real-time PCR (qRT–PCR), as previously described [23]. *EPSPS* and *ALS* primers were developed by Gaines et al. (2010) [12], and *CPS* primers were obtained from Ma et al. (2013) [54]. The relative *EPSPS or ALS* gene copy numbers were calculated using the comparative C_t_ method as 2^ΔCt^ (ΔC_t_ = C_t_^EPSPS/ALS^ − C_t_^CPS^) [55]. Controls containing water were included to check for contamination in the qRT-PCR reactions. *EPSPS* and *ALS* gene copy numbers were determined in six to 12 individual plants per population.

### 3.7. ALS Gene Sequencing

Mutations conferring resistance to ALS inhibitors in *A. palmeri* at positions Ala122, Pro197, Ala205, Trp574, Ser653, and Gly654, located in the CAD and BE domains, were analyzed in the gDNA of plants from all populations. Between four and nine individual plants per population were sequenced. Amplification of the CAD and BE domains was performed using two pairs of primers: CAD (forward: 5′-CGCCCTCTTCAAATCTCATC-3′, and reverse: 5′-CAATCAAAACAGGTCCAGGTC-3′) and BE (forward:5′-TGCTATTGGAGCTGCTGTTG-3′ and reverse: 5′-CCTTCTTCCATCACCCTCTG-3′. All primers were obtained from Torra et al. (2020) [32]. Sequencing of the purified amplified DNA fragments was conducted by STAB VIDA (Caparica, Portugal).

### 3.8. Statistical Analysis

For the dose–response bioassays with vertical plates, results are expressed as a percentage relative to the untreated control, which set at 100% for each population. For each population and herbicide, untreated and treated plants were compared using Student’s *t* test (*p* < 0.05), with asterisks indicating significant differences between treatments.

For the shikimate content, mean values were calculated from samples of different individual plants, and one-way ANOVA followed by Tukey’s HSD post hoc test (*p* < 0.05) were used to assess statistically significant differences between treatments within each population. Different letters within each population indicate significant differences between treatments.

One-way ANOVA and Tukey’s HSD post hoc tests (*p* < 0.05) were also used to assess statistically significant differences between the *EPSPS* or *ALS* gene copy number among populations. Different letters indicate significant differences between populations.

All statistical analyses and graph generation were performed using R version 4.3.2 (R Core Team, 2023).

## 4. Conclusions

This study provides new insights into the resistance profile of *A. palmeri* populations in Spain. Among the five evaluated populations from Aragon, four exhibited resistance to herbicides that inhibit amino acid biosynthesis, although none of them showed multiple resistance. The population from Bujaraloz (B) demonstrated moderate resistance to glyphosate, with a 12-fold amplification of the *EPSPS* gene compared to the other populations. In contrast, the populations from Albelda (A) and Tamarite de Litera (D) carried mutations at position Trp574 of the *ALS* gene in half of their individuals. In the population from Caspe (C), mutations at both Trp574 and Pro197 were detected in more than half of the individuals. This study shows the high genetic and morphological variability of this invasive weed and provides valuable information for correct IWM, promoting sustainable management. *Amaranthus palmeri* is establishing in Spain and is becoming a growing threat to agriculture. Of particular concern is the spread of resistant populations, such as those described in this study, especially considering the high selection pressure caused by frequent herbicide use in the region and the lack of alternative active ingredients, which will likely continue to promote resistance. Understanding the specific herbicide resistance mechanisms provides valuable information for making informed decisions about herbicide selection. However, the effective control of *A. palmeri* cannot rely exclusively on chemical methods, and non-chemical alternatives must be integrated into weed management programs. This approach ensures long-term effectiveness and minimizes the risk of further resistance development.

## Figures and Tables

**Table 1:** Analysis of the *ALS* sequencing in individuals of five populations of *Amaranthus palmeri* from Aragon (n = 4–9). In each population, the percentage of mutated and non-mutated individuals is shown. The amino acid codified in the mutated sequences is indicated in parenthesis (two in the case of heterozygous individuals).

	CAD Domain			BE Domain		
	Ala122	Pro197	Ala205	Trp574	Ser653	Gly654
**Population B**						
Non-mutated	100%	100%	100%	100%	100%	100%
**Population R**						
Non-mutated	100%	100%	100%	100%	100%	100%
**Population A**						
Non-mutated	100%	100%	100%	50%	100%	100%
Mutated				50% (Leu)		
**Population C**						
Non-mutated	100%	43%	100%	43%	100%	100%
Mutated		28.5% Thr 28.5% (Pro/Thr)		28.5% (Leu) 28.5% (Trp/Leu)		
**Population D**						
Non-mutated	100%	100%	100%	34%	100%	100%
Mutated				66% (Trp/Leu)		

**Table 2:** Location, year, and background of the five populations of *Amaranthus palmeri* from the provinces of Huesca (HU) and Zaragoza (ZA) in the region of Aragon (Spain) used in this study.

Population Code	Location	Coordinates	Collection Year	Site Background
**A**	Albelda (HU)	41.841621, 0.496103	2020	Crop field
**B**	Bujaraloz (ZA)	41.483769, 0.150913	2020	Roadside
**C**	Caspe (ZA)	41.235783, 0.054056	2020	Roadside
**D**	Tamarite de Litera (HU)	41.766663, 0.344205	2022	Crop field
**R**	Altorricon (HU)	41.771093, 0.376554	2021	Roadside

## Data Availability

The data are available upon request.

## Supplementary Materials

The following supporting information can be downloaded at: https://www.mdpi.com/article/10.3390/plants14101505/s1, Figure S1: Visualization of *Amaranthus palmeri* plants of population B 19 days after treatment. Each half-tank corresponds to one treatment (untreated (control) and glyphosate at the recommended field rate (1×; 0.84 kg ha^−1^) and three times the recommended field rate (3×; 2.52 kg ha^−1^)); Figure S2: Visualization of *Amaranthus palmeri* plants of population D 10 days after treatment with sulfonylurea or non-treated (control); Figure S3. Representative plates from the dose–response experiments used to select the glyphosate and nicosulfuron doses to be applied in the study. Table S1: Nucleotide sequence alignment and analysis of a portion of *ALS* gene sequence in *Amaranthus palmeri* populations from Aragon.

## Abbreviations

The following abbreviations are used in this manuscript:

ALS	Acetolactate synthase
CPS	Carbamoylphosphate synthetase
EPSPS	5-Enolpyruvylshikimate-3-phosphate synthase
IWM	Integrated Weed Management
NTSR	Non-target-site resistance
qRT-PCR	Quantitative real-time PCR
TSR	Target-site resistance
