# Supplementary material for: Resistance to Amino Acid Biosynthesis Inhibiting-Herbicides in Amaranthus palmeri Populations from Aragon (Spain)
Source: Plants (Basel). 2025 May 17;14(10):1505. doi: 10.3390/plants14101505 (PMC12115111; doi:10.3390/plants14101505)
Supplement: Supplementary file 1 [file plants-14-01505-s001.zip › plants-3610668-supplementary.pdf]

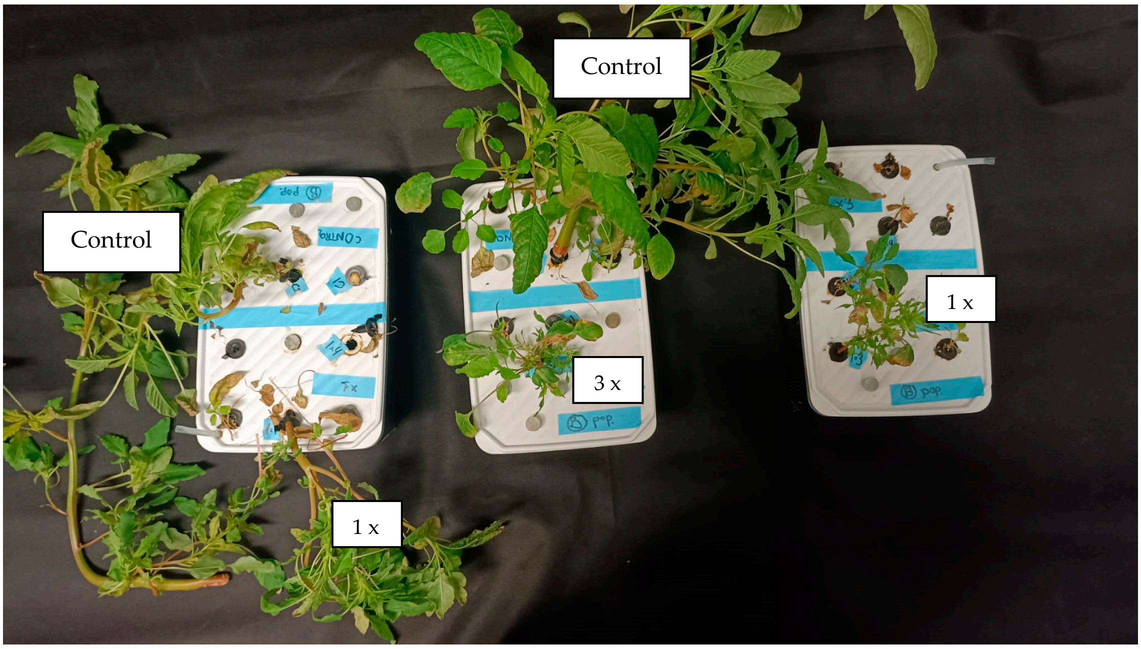


**Figure S1**. Visualization of *Amaranthus palmeri* plants of population B 19 days after treatment. Each half-tank corresponds to one treatment (untreated (control) and glyphosate at the recommended field rate (1×; 0.84 kg a.e ha$^{-1}$) and three times the recommended field rate (3×; 2.52 kg a.e. ha$^{-1}$).

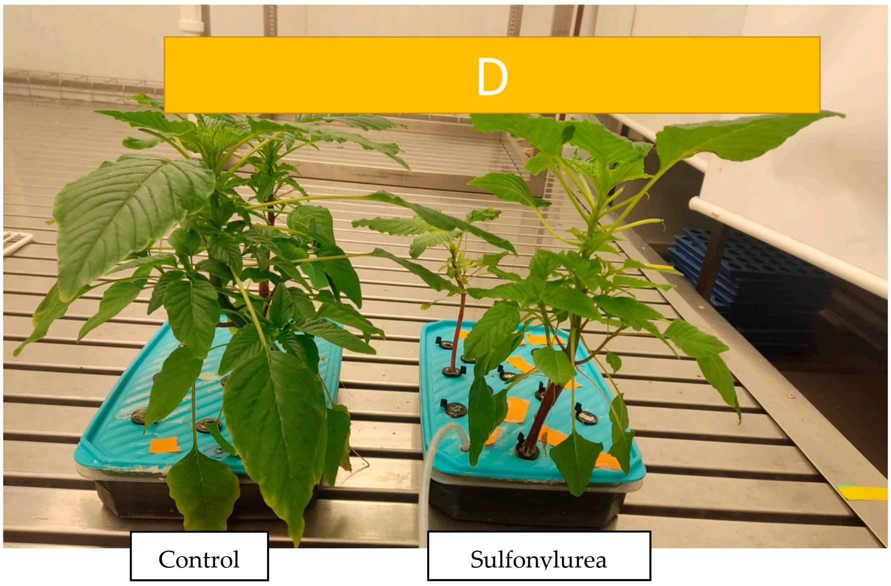


**Figure S2**. Visualization of *Amaranthus palmeri* plants of population D 10 days after treatment with sulfonylurea or non-treated (control).

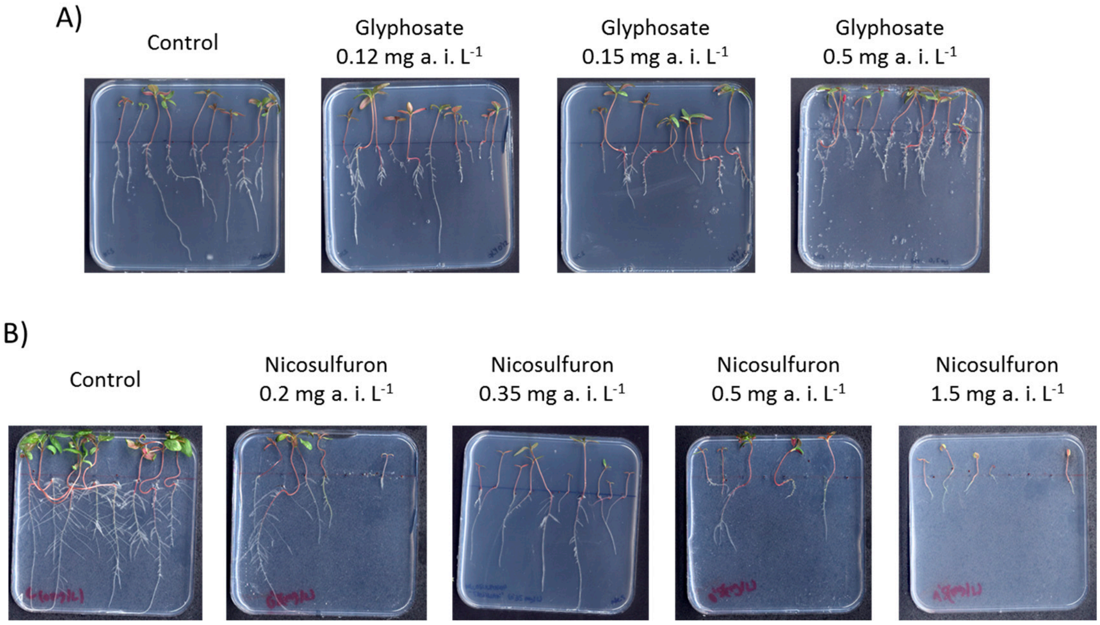


**Figure S3**. Representative plates from the dose-response experiments used to select the glyphosate and nicosulfuron doses to be applied in the study.

**Table S1**. Nucleotide sequence alignment and analysis of a portion of *ALS* gene sequence in *Amaranthus palmeri* populations from Aragon. Each line corresponds to a different individual plant. Positions Ala122, Pro197 and Ala205 are located in CAD domain CAD and Trp574, Ser 653 and Gly 654 appear in B domain of ALS gene sequence.

| | **Ala122** | **Pro197** | **Ala205** | **Trp574** | **Ser653** | **Gly654** |
|---|---|---|---|---|---|---|
| ALS A pop.1 | ..GCA.. | ..CCC.. | ..GCT.. | | | |
| ALS A pop.7 | ..GCA.. | ..CCC.. | ..GCT.. | **..TTG..** | ..CCT | AGC.. |
| ALS A pop.8 | ..GCA.. | ..CCC.. | ..GCT.. | | | |
| ALS A pop.9 | | | | ..TGG.. | ..CCT | AGC.. |
| ALS A pop.10 | ..GCA.. | ..CCC.. | ..GCT.. | | | |
| ALS B pop.11 | ..GCA.. | ..CCC.. | ..GCT.. | ..TGG.. | ..CCT | AGC.. |
| ALS B pop.12 | ..GCA.. | ..CCC.. | ..GCT.. | ..TGG.. | ..CCT | AGC.. |
| ALS B pop.13 | ..GCA.. | ..CCC.. | ..GCT.. | ..TGG.. | ..CCT | AGC.. |
| ALS B pop.14 | ..GCA.. | ..CCC.. | ..GCT.. | | | |
| ALS B pop.15 | ..GCA.. | ..CCC.. | ..GCT.. | ..TGG.. | ..CCT | AGC.. |
| ALS B pop.16 | ..GCA.. | ..CCC.. | ..GCT.. | ..TGG.. | ..CCT | AGC.. |
| ALS B pop.17 | ..GCA.. | ..CCC.. | ..GCT.. | ..TGG.. | ..CCT | AGC.. |
| ALS B pop.19 | ..GCA.. | ..CCC.. | ..GCT.. | | | |
| ALS C pop.22 | ..GCA.. | ..CCC.. | ..GCT.. | ..TGG.. | ..CCT | AGC.. |
| ALS C pop.25 | ..GCA.. | **..ACC..** | ..GCT.. | ..TGG.. | ..CCT | AGC.. |
| ALS C pop.26 | ..GCA.. | ..**ACC**.. | ..GCT.. | ..TGG.. | ..CCT | AGC.. |
| ALS C pop.27 | ..GCA.. | ..CCC.. | ..GCT.. | **..TTG..** | ..CCT | AGC.. |
| ALS C pop.28 | ..GCA.. | ..CCC.. | ..GCT.. | **..TTG..** | ..CCT | AGC.. |
| ALS C pop.29 | ..GCA.. | ..**A/CCC**.. | ..GCT.. | **..TT/GG..** | ..CCT | AGC.. |
| ALS C pop.30 | ..GCA.. | ..**A/C**CC.. | ..GCT.. | **..TT/GG..** | ..CCT | AGC.. |
| ALS D pop.C2 | ..GCA.. | ..CCC.. | ..GCT.. | | | |
| ALS D pop.C3 | ..GCA.. | ..CCC.. | ..GCT.. | **..TT/GG..** | ..CCT | AGC.. |
| ALS D pop.T2 | ..GCA.. | ..CCC.. | ..GCT.. | **..TT/GG..** | ..CCT | AGC.. |
| ALS D pop.T3 | ..GCA.. | ..CCC.. | | ..TGG.. | ..CCT | AGC.. |
| ALS R pop.31 | ..GCA.. | ..CCC.. | ..GCT.. | ..TGG.. | ..CCT | AGC.. |
| ALS R pop.32 | ..GCA.. | ..CCC.. | ..GCT.. | | | |
| ALS R pop.33 | ..GCA.. | ..CCC.. | ..GCT.. | ..TGG.. | ..CCT | AGC.. |
| ALS R pop.34 | ..GCA.. | ..CCC.. | ..GCT.. | ..TGG.. | ..CCT | AGC.. |
| ALS R pop.35 | ..GCA.. | ..CCC.. | ..GCT.. | ..TGG.. | ..CCT | AGC.. |
| ALS R pop.36 | ..GCA.. | ..CCC.. | ..GCT.. | ..TGG.. | ..CCT | AGC.. |
| ALS R pop.38 | ..GCA.. | ..CCC.. | ..GCT.. | ..TGG.. | ..CCT | AGC.. |
| ALS R pop.39 | ..GCA.. | ..CCC.. | ..GCT.. | | | |
| ALS R pop.40 | ..GCA.. | ..CCC.. | ..GCT.. | ..TGG.. | ..CCT | AGC.. |
